# Human stem cell models of dementia

**DOI:** 10.1093/hmg/ddu302

**Published:** 2014-06-16

**Authors:** Frederick J. Livesey

**Affiliations:** Gurdon Institute, Cambridge Stem Cell Institute & Department of Biochemistry, University of Cambridge, Tennis Court Road, CambridgeCB2 1QN, UK

## Abstract

Positive predictions were made in the aftermath of the development of induced pluripotent stem cell technology for the use of patient-specific iPSCs to model neurological diseases, including dementia. Here, we review the current state of the field and explore how close we are to the goal of *in vitro* models that capture all aspects of the cell and molecular biology of dementia.

## INTRODUCTION

There are an estimated 6 million people with dementia in Europe, and 44 million worldwide (Alzheimer's Disease International; www.alz.co.uk). Alzheimer's disease (AD) is the commonest cause of dementia and accounts for over 60% of cases, yet there are currently no licensed drugs that modify the course of the disease. Aside from vascular dementia, the majority of non-Alzheimer-type dementia is accounted for by Lewy body dementia, frontotemporal dementia (FTD), Parkinson's disease dementia (PD dementia) and a group of less common conditions that includes progressive supranuclear palsy and corticobasal degeneration.

A challenge for modelling dementia, and developing therapies based on those models, is our incomplete understanding of the cell and molecular biology underlying the initiation and progression of the disease. Animal models continue to be critical to understanding the pathogenesis of AD, for example. A number of different transgenic mice expressing human AD causing mutations in single genes have been generated, most notably using the human *MAPT*/*tau*, *amyloid precursor protein* (*APP*) and *PSEN1* genes (1). Those animals develop many different aspects of the AD phenotype, although there are notable gaps, including, for example, the absence of neuronal loss in many models and the difficulty in generating neurofibrillary tangles in mice without *MAPT* mutations (1). It is clear that no animal model completely models AD and there is an ongoing need for tractable systems for studying AD pathogenesis both *in vitro* and *in vivo*.

Advances in techniques for generating stem cells, genome engineering and the generation of neural cell types are making the production of human neural circuits *in vitro* relatively routine (2). Combining these approaches with monogenic forms of dementia has raised the prospect of creating human cellular models of the initiation and progression of dementia in the cell types affected by the disease. However, while the initial reports of the development of dementia-relevant phenotypes in human stem cell-derived neurons are promising, it is useful to take stock of the scale of the challenge of modeling a complex, late-onset disease *ex vivo*.

An idealized *in vitro* model of dementia, as with any disease, would recapitulate the disease process from initiating events through disease progression, including neuronal and synaptic dysfunction, to neurodegeneration and neuronal cell death. It would include all contributing cell types and would reflect *in vivo* biology in all aspects with one exception: disease onset and progression would be fixed, predictable and over a significantly abbreviated timescale, so as to be amenable to experimental investigation. This review will summarize the current state of the field and explore whether the longer-term goal of *in vitro* models that capture all aspects of dementia is realistic and achievable.

## MAKING HUMAN FOREBRAIN NEURONS AND NEURONAL CIRCUITS

Is it necessary to generate the particular types of neurons affected by dementia? This question relates to the classic pathology of selective neuronal vulnerability and is not unique to dementia. Related questions are whether, using completely penetrant, genetic forms of disease, neurodegenerative diseases such as AD can initiate in any neuronal type, and also whether there are specific cell types that are particularly vulnerable to disease progression or spread through the nervous system. Stem cell systems allow these and related questions to be addressed, assuming that these systems can recapitulate disease pathogenesis.

The major brain regions affected by dementia are the cerebral cortex and hippocampus. A number of different approaches to generating patient-specific cortical neurons have been developed in recent years. Directed differentiation of pluripotent stem cells (PSCs) generates all classes of cortical projection neurons over several weeks, replaying *in vivo* cortical development in cell culture (3,4). Similar approaches have been developed to generate hippocampal neurons (5). Transdifferentiation of dermal fibroblasts directly to cortical neurons is a promising approach (6), although this method has not been reliably used for disease modeling in AD to date (see retraction notice to (7)). A hybrid approach, driving differentiation of iPSCs to upper layer cortical neurons using similar transcription factor combinations as used for transdifferentiation, combines many of the positive features of the two other approaches: iPSCs are a renewable resource, so neuronal numbers are not limited, and the process takes less time than directed differentiation to generate mature neurons (8).

Given that many of the major diseases of the cerebral cortex, including AD, are diseases of synaptic function, one desirable objective is to generate cortical networks *in vitro* that resemble those found *in vivo.* To date, the functional properties of neural networks formed by PSC-derived neurons have not been extensively studied (9). Up to now, efforts have been concentrated on the production of specific cell types rather than on developing representative neural networks, although this is rapidly changing. This is an important issue for the future exploitation of stem cell models for functional studies of synaptic function in dementia and also for the potential generation of models of dementia progression through the central nervous system.

One pressing question is whether it is possible in two-dimensional culture for neural networks to form that are representative of the specific wiring observed *in vivo*. It may be that cortical lamination in three-dimensional systems is essential for accurate micro-circuit formation. Recent work in the generation of 3D human cortical organoids is promising (10,11), although there are technical hurdles that need to be overcome to allow production of all classes of neurons in these systems, as well as full lamination to take place.

In addition to models of local cortical circuits, models to study dementia initiation and progression in the context of different human neural circuits, including corticocortical, corticothalamic and corticohippocampal, would be very useful for functional studies of AD pathogenesis and testing of therapeutic intervention strategies. The development of optogenetic and chemogenetic techniques to control neuronal firing (12), combined with advances in genome engineering in stem cells (13), offers the potential for accurate control of circuit inputs, activity and outputs in healthy and AD-affected circuits.

## STEM CELL MODELS OF GENETIC FORMS OF ALZHEIMER'S DISEASE

**Figure 1.**
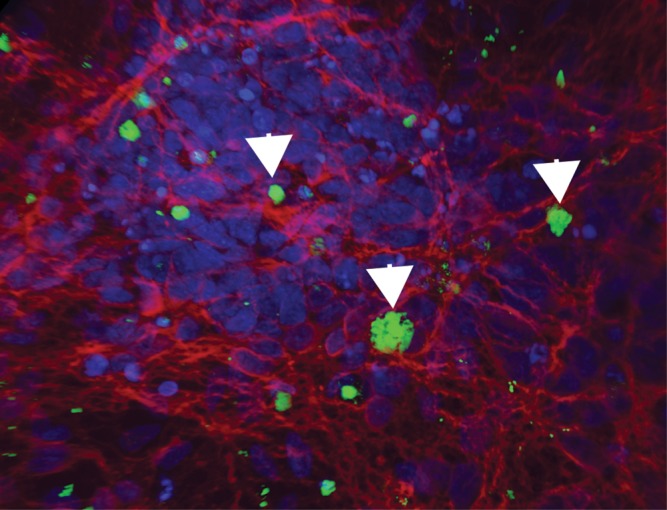
Aggregation of insoluble Aβ42 in cultures of Trisomy 21 neurons. Large numbers of intracellular and extracellular deposits of Aβ42 are found in cultures of Trisomy 21/Down syndrome iPS cell-derived cortical neurons between 60 and 90 days of culture.

Monogenic, familial AD (FAD) is an autosomal dominant, completely penetrant and early onset disease (14). All known FAD mutations are located in the *APP* locus or the catalytic components to the γ-secretase complex that processes APP, encoded by presenilin (*PSEN*)-1 and -2 (15). These mutations account for <1% of all AD and strongly implicate altered APP processing as the key initiating event in the disease (15). Early onset AD also occurs in individuals with a duplication of the *APP* locus, a rare cause of FAD (16), and in individuals with Trisomy 21/Down syndrome (17), suggesting that increasing *APP* dosage is sufficient for disease initiation (16) (Fig. 1).

In recent years, several groups have reported generation of FAD iPSCs and disease-related phenotypes in neurons derived from those iPSC lines. Labs have tended to concentrate on mutations in either *PSEN1*, the catalytic subunit of γ-secretase, or in *APP*, and a common feature of all reports are changes in APP processing and amyloid β (Aβ) peptide production.

In the first publication modeling FAD, Yagi and colleagues generated iPSCs carrying mutations in *PSEN1* and *PSEN2* (18). Neurons generated from those iPSCs had changes in the Aβ40 : Aβ42 ratio, with increased production of Aβ40 and Aβ42 peptides. However, this phenotype varied among lines and was not statistically different compared with peptide production control cells (18). A recent report on a different set of *PSEN1* mutations reported changes in Aβ peptide production at the neural progenitor stage, with an increase in Aβ42 relative to Aβ40 (decreased Aβ40 : Aβ42 ratio) (19). In addition, genome engineering has been used to produce an allelic series of deletions in exon 9 of *PSEN1*, demonstrating that this deletion reduces PSEN1 protein function, without generating null alleles (20).

Investigations into *APP* mutations have studied the effects of *APP* duplication, point mutation and deletions. Analyses of neurons generated from *APP* duplication iPSCs identified an increase in Aβ40 production, compared with healthy controls (21). However, extracellular Aβ42 levels were lower than detectable levels in that system (21). Israel and colleagues observed an increase in tau phosphorylation in *APP* duplication neurons. Pharmacological inhibition of γ- or β-secretase inhibition both prevented the increased production of Aβ40, however, only β-secretase inhibition also prevented increased tau phosphorylation (21). Similarly, Trisomy 21/Down syndrome neurons, in which there is an additional copy of the *APP* gene (22), were found to increase production of Aβ peptides, which lead to the accumulation of Aβ in extracellular aggregates (23). Trisomy 21/Down syndrome neurons also demonstrated hyperphosphorylation of tau and redistribution to the somatodendritic compartment of forebrain neurons (23).

Analyses of neurons generated from iPSCs carrying the recessive E963Δ and dominant V717L mutations in *APP* identified notable differences in phenotypes: whereas the V717L mutation led to a decrease in the Aβ40 : Aβ42 ratio, the E963Δ mutation led to an overall reduction in extracellular Aβ peptide production, and an increase in the accumulation of intracellular Aβ oligomers (24). Detailed study of neurons generated from *APP* V717I iPSCs also identified a decrease in the Aβ40 : Aβ42 ratio, and also observed an increase in total and phosphorylated tau (25).

What these papers have in common is a focus on disease initiation, and particularly APP processing to generate Aβ peptides. It is reassuring that cellular phenotypes that are a direct consequence of the specific mutations are relatively easily observed in culture, most notably changes in Aβ peptides. Although several papers reported changes in tau expression, phosphorylation and cellular localization in different models, there is still progress to be made on exploring the link between APP processing and tau changes in human neurons. While the systems used to date are simplified to neurons and astrocytes, they provide a powerful platform to address the key proposals of the amyloid hypothesis, the current dominant theory for the mechanisms underpinning AD initiation and progression (26).

The amyloid hypothesis for AD initiation was formulated based on the discovery that genetic forms of the disease are due to changes in genes that are involved in proteolyic processing of the single-pass transmembrane APP to generate short Aβ peptides (27). The amyloid hypothesis proposes that accumulation of the longer, 42 amino acid form of Aβ, Aβ42, is the key initiating event in AD, which subsequently leads to changes in neuronal function and ultimately cell death (27). A critical second step in AD progression is mediated by changes in the posttranslational modifications and cellular localization of the microtubule-associated protein tau, which proceeds to form insoluble intracellular aggregates termed neurofibrillary tangles (28,29). If stem cell systems do develop disease-relevant tau changes, clearly they provide a useful platform to test the amyloid hypothesis.

## MODELING SPORADIC, LATE-ONSET ALZHEIMER'S DISEASE

Late-onset AD makes up over 99% of cases of AD and is highly heritable, estimated at 60–70% (30). A pressing question is how to model sporadic AD *in vivo* or *in vitro*. Genome-wide association studies in sporadic AD have identified one major susceptibility gene, the ε4 allele of ApoE, and several additional susceptibility loci, including PICALM, BIN1, SORL1, Clusterin/ApoJ and CR1 (31). More recently, disease-associated variants in TREM2 (32,33) and a coding variant in APP which protects individuals from AD (34) have been identified.

Models of sporadic AD would provide an opportunity to compare the biology of monogenic forms of the disease with the more common sporadic form, as well as being useful tools to study AD initiation and progression. Two studies have asked whether stem cell models generated from patients with sporadic AD will develop AD phenotypes in culture (21,24). In both cases, as well as generating models from genetic forms of AD, models were also developed from two sporadic AD patients. Strikingly, in each report neurons from one patient developed disease-relevant phenotypes and neurons from the other did not (21,24). While these numbers are not sufficiently large to draw meaningful conclusions, the significant heritability of AD does suggest that modelling sporadic AD is feasible, if only in the subset of patients with the most significant genetic contribution to disease initiation.

One key aspect of AD biology that has not yet been captured in stem cell models is the role of inflammation, and specifically of microglia. An advantage of stem cell systems is the ability to generate different cellular components of differing genotypes for co-culture to address the contribution of each to disease initiation and progression. Methods have been developed to generate microglia from mouse PSCs (35), which are likely to be similarly applicable to human PSCs. The modular nature of stem cell systems is ideal for assembling models that combine neuronal initiation of disease with astrocytes and microglia of different, disease-associated genotypes to investigate the functional contribution of each cell type and genotype to disease progression.

## STEM CELL MODELS OF NON-AD DEMENTIA

In addition to investigating Alzheimer-type dementia, there have been several studies of genetic forms of non-Alzheimer-type dementias, including FTD due to polymorphisms or mutations in *MAPT*/tau, *Progranulin* and *C9ORF72* (36–38). Again, a common theme here is that some, but not all, aspects of the cell and molecular biology directly downstream of the genetic lesion were detected in neurons in culture. For example, neurons carrying the expanded hexanucleotide repeat in *C9ORF72* developed both RNA foci and accumulated of RAN peptides, as found *in vivo* (37,39,40). However, as with studies of FAD, novel biological insights have not yet been derived from these proof of concept models.

## FUNCTIONAL MATURITY VS CELLULAR AGEING

For neurons that are generated by directed differentiation from iPS or ES cells, the process recapitulates *in vivo* development at many different levels, including neuronal maturation (4). A consequence of using developmental biology to generate neuronal types is that human cellular systems take an inherently long period of time to acquire functional maturity, compared with their rodent equivalents (4,41,42). Methods are currently being developed to accelerate the entire differentiation process, including neuronal maturation, using small molecules in place of recombinant proteins to more efficiently manipulate cell signaling systems (43).

A recurring theme, related to the question of cellular maturity, is how a disease process that takes decades to become clinically obvious *in vivo* can reasonably be expected to replay over months *in vitro*. Given that the single greatest risk factor for the development of neurodegeneration is age, there is considerable interest in whether it is possible to induce cellular ageing in culture (44). Recent work from the Studer lab used forced expression of Progerin, a pathogenic variant of Lamin A that is responsible for Hutchinson-Gilford progeria (45), to induce ageing in midbrain neurons (46). This approach induced the cellular hallmarks of ageing and led to more pronounced Parkinson's disease phenotypes in midbrain neurons (46).

## CONCLUSIONS AND OUTLOOK

A considerable body of work over recent years has established that human neurons generated from iPSCs carrying dementia-causing mutations develop cellular phenotypes that are direct consequences of those mutations. However, it could be argued that these systems have not yet delivered a novel biological insight into the mechanisms of disease initiation and progression, let alone one that has been tested *in vivo.* There appears to be considerable potential for stem cell systems to enable experimental work on the basic biology of dementia. Current limitations include the limited characterization of the neurobiology of these systems, including the extent to which the neural networks formed *in vitro* are representative of those operating *in vivo*. Furthermore, the relative youth of the neurons derived from iPSCs, and its relative importance for modeling adult onset disease, are becoming widely appreciated. However, these are not insurmountable challenges, and the first wave of reports are encouraging for the utility of stem cell systems for both modeling and understanding the cell and molecular biology of dementia in human neuronal systems.

## FUNDING

F.J.L. is a Wellcome Trust Senior Investigator. Research in his group is supported by the Wellcome Trust, Alzheimer's Research UK, EU IMI StemBANCC and the Medical Research Council and Cancer Research UK. Funding to pay the Open Access publication charges for this article was provided by the Wellcome Trust.
